# Wheat transcriptomic responses to extended feeding by wheat curl mites

**DOI:** 10.1038/s41598-022-16792-1

**Published:** 2022-07-22

**Authors:** Lise Pingault, Tran Kim Ngan Luong, Joe Louis, Gary Hein

**Affiliations:** 1 Department of Entomology, University of Nebraska-Lincoln, Lincoln, NE 68583 USA; 2 Department of Biochemistry, University of Nebraska-Lincoln, Lincoln, NE 68583 USA

**Keywords:** Plant immunity, Plant stress responses

## Abstract

The economic importance of wheat and its contribution to human and livestock diets has been already demonstrated. However, wheat production is impacted by pests that induce yield reductions. Among these pests, wheat curl mite (WCM, *Aceria tosichella* Keifer) impacts wheat all around the world. WCM are tiny pests that feed within the whorl of developing leaves, and their feeding causes leaf curling by preventing them from unfurling. The curling of the leaves provides a protective niche for the WCM. Additionally, WCM are also the vector of serious viruses in wheat. Little is known regarding the impact of the WCM on wheat transcriptome, and to date, only one article has been published describing the wheat transcriptomic changes after 1 day of WCM feeding. To better understand the wheat transcriptome variation after extended feeding by WCM [10 days post infestation (dpi)], we used an RNA-seq approach. We collected WCM-infested and uninfested leaves from two wheat cultivars: Byrd (WCM resistant) and Settler CL (WCM susceptible) at 10 dpi. Our transcriptomic analysis revealed the common and specific transcriptomic variations in WCM resistant and susceptible wheat cultivars, chromosome 3D specific location of the differentially expressed genes with functions involved in defense and stress response, and also identified the gene functions related to lipid signaling and membrane integrity, and phytohormone pathways potentially contributing to WCM resistance. Collectively, our study provides important insights on wheat defense mechanisms against WCM after extended feeding.

## Introduction

Wheat (*Triticum aestivum* L.) is one of the most crucial crops worldwide, contributing significantly to human food security. Wheat production is affected by many different pests; however, the wheat curl mite (WCM), *Aceria tosichella* Keifer, is one of the most economically significant global pests of wheat. When the microscopic WCM (ca. 0.2 mm long) arrives on a wheat plant, it moves to the base of the newest leaf developing within the whorl and begins feeding^1^. WCM feeds on the epidermal tissues in the grooves between leaf veins, creating damage to bulliform cells^2^. This feeding impacts the ability of the leaves to unfurl. Mite-infested leaves tend to have their edges curled tightly toward the mid-rib, and the tips of new leaves can become trapped in this rolled leaf forming a loop^1–3^. The whorl and curled leaves provide WCM a more humid micro-environment beneficial for survival and reproduction, and shelter from miticidal exposure. WCM feeding damages plants by withdrawing the nutrients and distorting leaf growth, thus reducing photosynthesis and respiration^4^. Direct feeding from large populations of WCM can result in ~ 15% yield loss^5^.

The main impact of WCM results from their ability to transmit viruses to wheat. In North America, WCM is the only known vector of *Wheat streak mosaic virus*^3^, *High Plains wheat mosaic virus*^6^, and *Triticum mosaic virus*^7^. While co-infections are common^8,9^, significant impact on wheat yield and quality can result from the presence of only one or more viruses in the disease complex^7,8^. WCM reproduces rapidly. With temperature between 23 and 27 °C, a new generation can develop every 8 to 10 days^10^. Mites disperse via wind currents, increasing their ability to spread viruses. The presence of volunteer wheat plays a significant role in the survival and spread of WCM and the epidemiology of viruses in winter wheat^1^. Volunteer wheat, especially that emerging before wheat harvest, provides a ‘green bridge’ to sustain the WCM between summer harvest and the emergence of the new crop in the fall^11,12^. Current management strategies for this wheat-mite-virus complex focus on reducing the impact of ‘green bridge’ hosts, adjusting planting date, and resistant wheat varieties^13,14^.

Understanding the wheat-mite-virus complex is challenging because WCM is a cryptic species complex^15^. In North America, two *Aceria tosichella* haplotypes have been identified (Type 1 and Type 2) based on their genetic differences of mitochondrial DNA cytochrome oxidase I and II (COI and COII) and ribosomal DNA internal transcribed spacer 1 (ITS1)^16^. Biological differences between these WCM genotypes have been shown for wheat virus transmission efficiencies^12,17–19^, reproductive ability on virus-infect plants^20^, effects of temperature on population growth rates^21^, as well as a differential response to several mite resistant genes in wheat^22,23^.

Historically, wheat has not been found to possess significant resistance against the WCM^24^. This led to efforts to identify and develop resistance genes from close relatives of wheat. To date, four different curl mite colonization (*Cmc*) genes have been identified, chronologically *Cmc3*, *Cmc1*, *Cmc2*, and *Cmc4*^25^. *Cmc3* was translocated from rye (*Secale cereale* L.) to chromosome arm 1AL of wheat and released commercially as ‘TAM107’^26,27^. However, the extensive planting of ‘TAM107’ during the 1980’s into the mid 1990’s led to WCM adaptation and loss of effectiveness of the gene^17,28^. *Cmc2* was found in *Agropyron elongatum* (Host) Beauv. and translocated to chromosome arm 6DL of wheat^29^, but there has been no further development. *Cmc1* was transferred from *Aegilops tauschii* (Coss.) Schmal to chromosome arm 6DS of wheat^29,30^. *Cmc1* is a single dominant resistance gene and was used to develop breeding material with a variety release^31^. *Cmc4* was transferred from *Ae. tauschii* and found to also be on the short arm of chromosome 6D in wheat, but despite being on the chromosome 6DS in wheat, *Cmc1* and *Cmc4* were found to be independent^27^. Mite resistance has been found in the variety ‘Byrd’ that originated from one of it parents ‘TAM 112’^32^. Recently, the *Cmc* gene in TAM112 was mapped in the chromosome 6DS, similar to *Cmc4*^33^. Haplotype analysis using TAM112 suggests that the *Cmc* gene in TAM112 and *Cmc4* are the same gene^34^.

WCM resistance has great value in controlling the disease complex in the growing crop, but also by reducing mite buildup in volunteer wheat making up the summer ‘green bridge’. Recent development of effective virus resistance genes in wheat (*Wsm1*, *Wsm2*, *Wsm3*)^19,35,36^ also alter the importance of WCM. As the severe impact of the virus lessens with more virus-resistant wheat, the ability of WCM to build up to a large population becomes more significant^37^.

While WCM resistance genes have been growing in number, the interactions between wheat’s defense mechanisms and WCM’s response and adaptation to these genes is still largely unknown. Kiani et al.^38^ have provided the only study so far to identify potential genes and pathways in defense against WCM herbivory. After 24-h post infestation (hpi), TAM112 wheat plants showed modifications in their transcriptomes through the expression of genes involved in jasmonic acid (JA) defense pathways, WKRY transcription factors, antioxidation processes, and pathogen-related responses. However, these genes were unaffected in the WCM-susceptible variety ‘Karl 92’.

With evidence of WCM adaptation to *Cmc3*^6,22,28^, the stability and long-term efficacy of these defense mechanisms is a concern. Different WCM haplotypes have varied reactions to different resistance genes^22,27^. Moreover, the rapid reproductive rate of WCMs provides long-term advantages to mite populations in overcoming antibiotic-based resistance. For the development and effective deployment of a strategy for these resistance genes, it is important to know the plant-mite interactions and the categories of resistance involved. The goal of this study is to explore the transcriptome-level responses of wheat varieties with mite-resistant genes to continued mite feeding and the exposure of subsequent mite generations to plant defenses resulting from extended mite infestation. Results from this research will provide further insight into the interactions between resistant wheat varieties and WCMs, and propose more effective deployment strategies for this management tactic.

## Results

### Wheat transcriptomic responses to extended feeding by WCM

**Figure 1:**
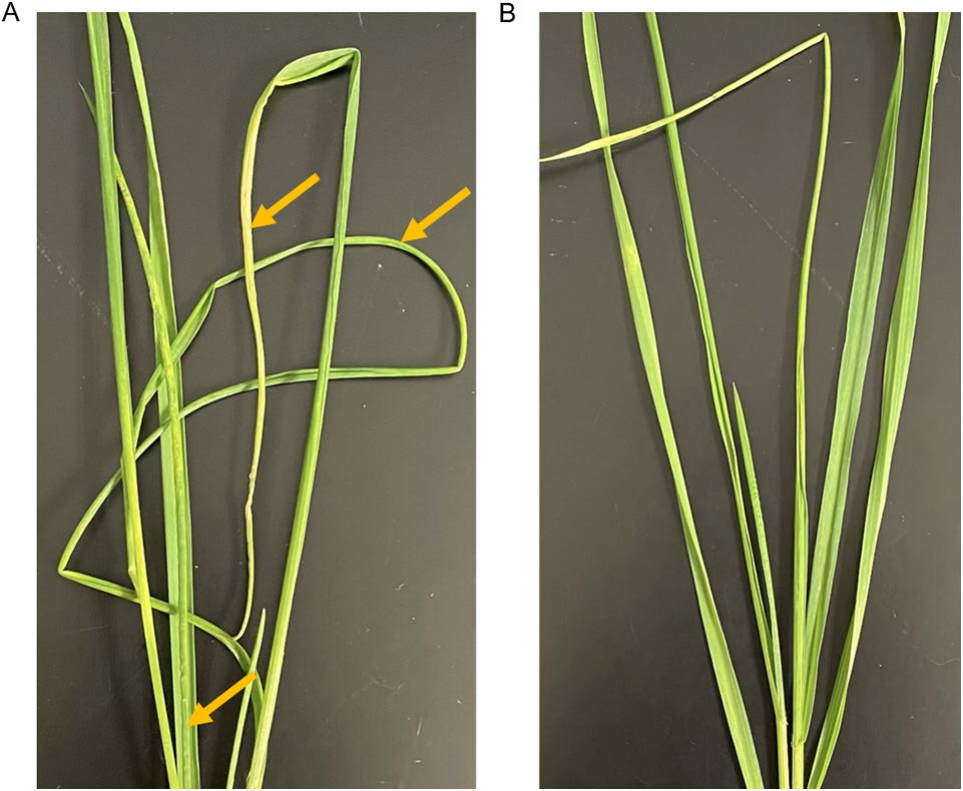
Leaf curling resulting from WCM feeding at 20 dpi in Settler CL (**A**) and Byrd (**B**). Arrows indicate curling locations on Settler CL leaves. The picture was taken by Tran Kim Ngan Luong.

For this study, two wheat varieties were selected because of their susceptibility (Settler CL) or resistance (Byrd) to WCM. Twenty days post infestation (dpi), wheat leaves experienced a different morphology for each variety. Leaf curling was observed for the susceptible variety (Fig. 1A), while the leaves of the resistant variety remained flat (Fig. 1B). To further investigate the underlying mechanisms of wheat responses against WCM, transcriptomic profiles of WCM-infested and uninfested control plants was performed at 10 dpi.

**Figure 2:**
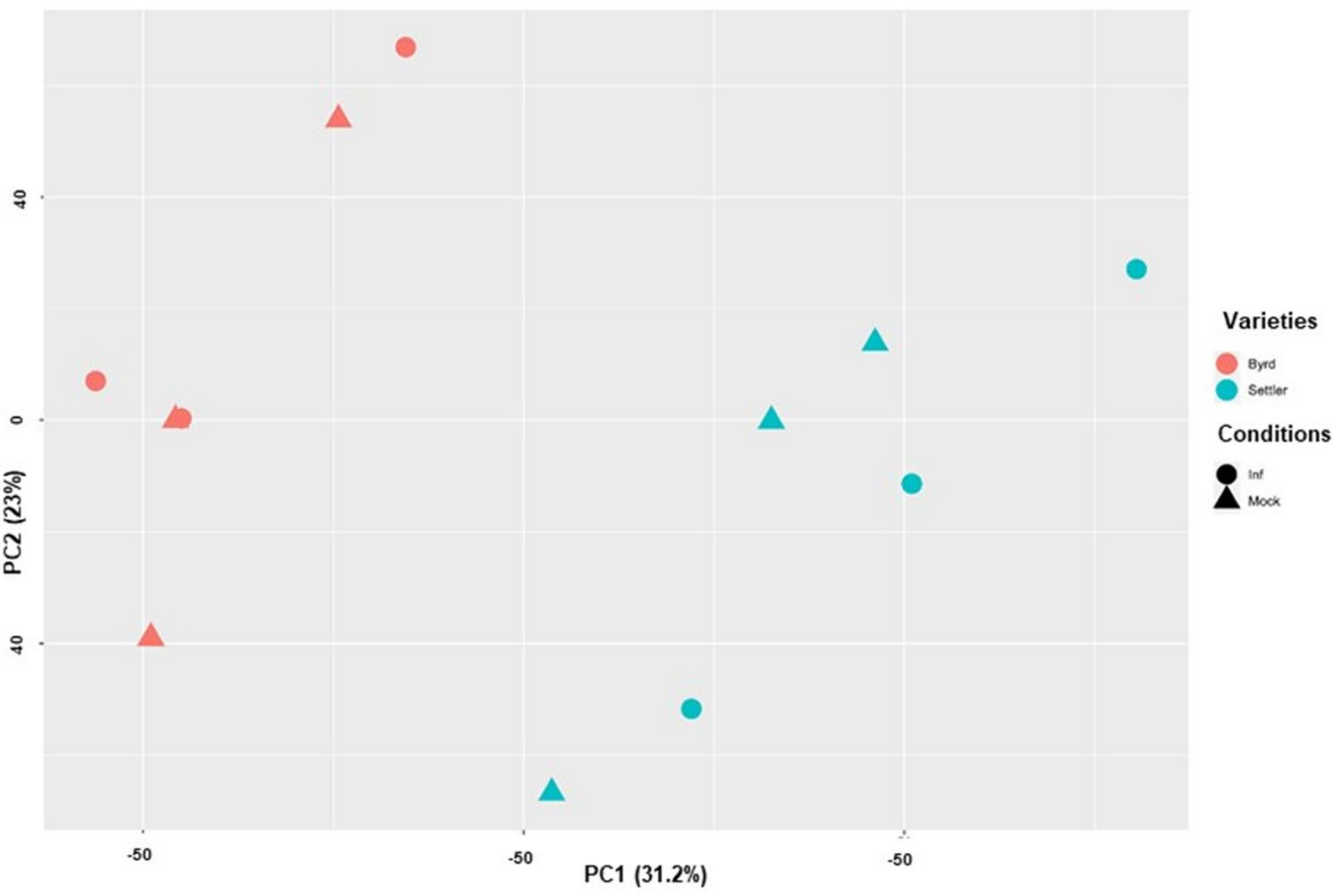
PCA analysis of the 106,817 high confidence genes expressed in at least one condition. Wheat cultivar is represented with different colors (red = Byrd and blue = Settler CL) and treatment condition with different shapes (circle = Infested and triangle = control).

The sequencing of the RNA-seq libraries for the two varieties at 10 dpi (WCM-infested and uninfested control) generated 20.7 million paired-end reads on average (Supplemental Table 1). Reads were mapped on the reference genome v2.1 of the variety Chinese Spring^39^, and an average of 17.2 million paired-end reads (83%) were mapped on the reference genome assembly (Supplemental Table 1). The PCA analysis was run for the 106,817 genes expressed in at least one condition, and responses for the two varieties separated in different groups (PC1, 31.2%) (Fig. 2). However, there was not a clear distinction between the infested or control conditions (PC2, 23%) (Fig. 2).

**Figure 3:**
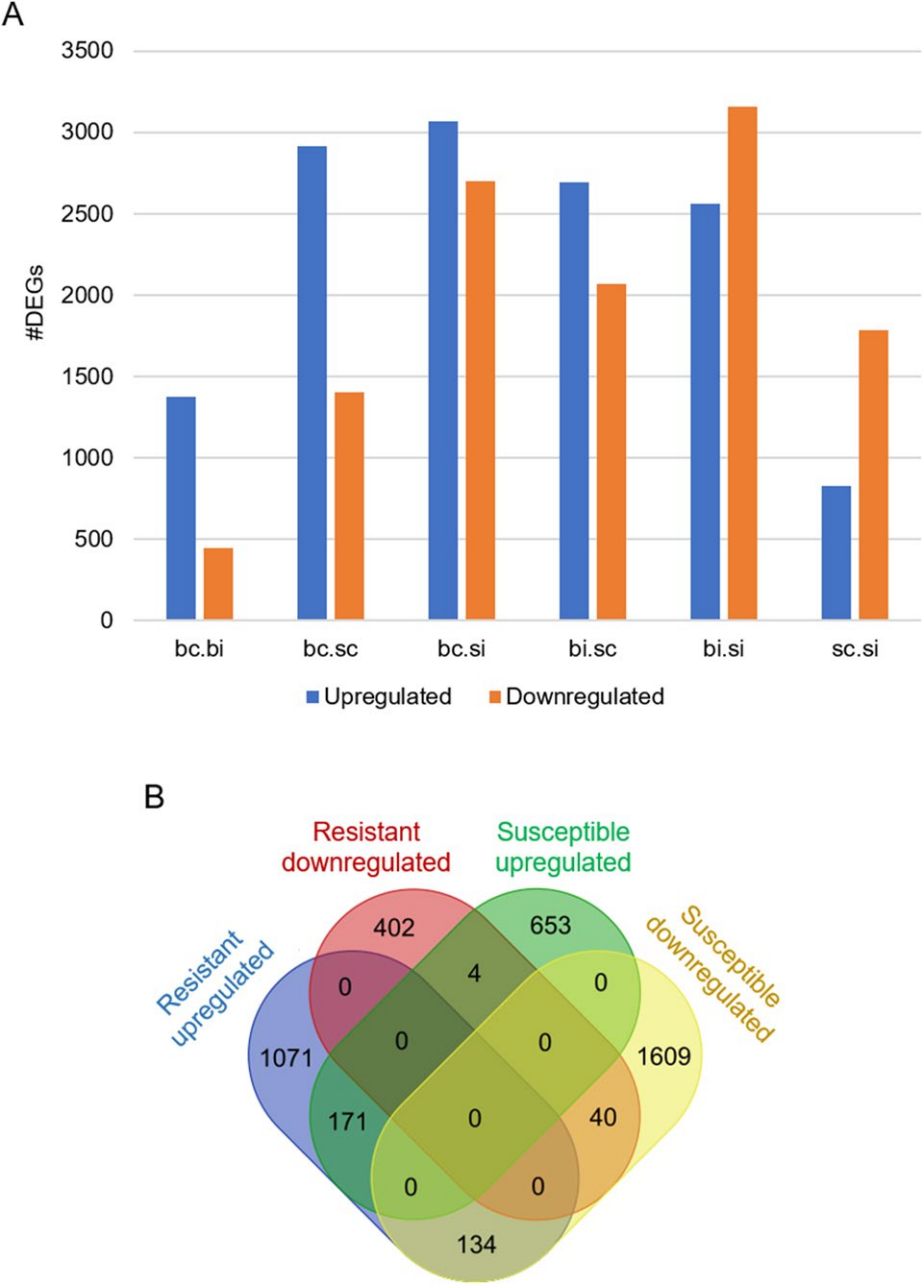
Overview of the 11,016 DEGs. (**A**) Partitioning of the DEGs as up or downregulated for all the comparisons. bc, Byrd control; bi, Byrd infested; sc, Settler CL control; si, Settler CL infested. (**B**) Venn diagram representing the overlap of the up and downregulated DEGs between all the comparisons.

The number of differentially expressed genes (DEGs) were characterized for the following comparisons: Byrd control versus Byrd infested, Byrd control versus Settler CL control, Byrd control versus Settler CL infested, Byrd infested versus Settler CL control, Byrd infested versus Settler CL infested and Settler CL control versus Settler CL infested, with the following parameters: |FC|> 2 and *P*-value < 5% (Supplemental Table 2). In total 11,016 non-redundant DEGs were identified. The number of genes up or downregulated for each comparison is shown in Fig. 3A. Among the 1822 DEGs in the resistant genotype, 75.5% (1376 genes) were upregulated at 10 dpi. By comparison, 2611 genes were differentially expressed in the susceptible genotype, including 31.7% (828 genes) upregulated genes at 10 dpi (Fig. 3A). Comparing the uninfested condition of the two varieties, 4322 genes were differentially expressed with 67.5% (2917 genes) upregulated genes in Settler CL. After infestation, 5717 genes were differentially expressed between both varieties and 55.2% (3154 genes) of the genes were upregulated in Byrd.

The overlap of the 4084 DEGs up or downregulated for the Byrd and Settler CL comparisons of control and infested conditions was represented with a Venn diagram (Fig. 3B). One hundred seventy-one genes were commonly upregulated after WCM infestation and 134 were commonly differentially expressed between the conditions upregulated in the resistant variety and downregulated in the susceptible variety after WCM infestation (Fig. 3B).

The function of the DEGs was impacted differently for each variety. In the resistant variety, lipid transport, lipid localization, or sugar metabolic process functions were downregulated (Supplemental Table 3). Alternatively, upregulated genes in the resistant variety were related to immune response, immune system process, and regulation of defense response functions (Supplemental Table 3). On the other hand, downregulated genes in the susceptible variety were related to positive regulator of stomatal complex development, tissue development, plant epidermis development, or polysaccharide catabolic process functions (Supplemental Table 3). Upregulated genes of the susceptible cultivar were related to metal ion transport, cellular localization, or defense functions (Supplemental Table 3).

### Downregulation of the genes located on the telomeric part of the chromosome 3DL of the susceptible wheat variety

Hexaploid wheat is composed of three sub-genomes: A, B and D. Version 2.1 of the annotation, which was used in our analysis, displayed the repartition of the protein coding genes equally among the 3 sub-genomes, A: 35,345 genes, B: 35,643 genes, and D: 34,212 genes^39^. Here, we further observed the repartition on the 21 wheat chromosomes of the 4084 DEGs in Byrd and Settler CL infested plants and their respective control (described in Fig. 3B). The highest number of DEGs were located on chromosome 3D (501 DEGs) (Supplemental Fig. 1).

**Figure 4:**
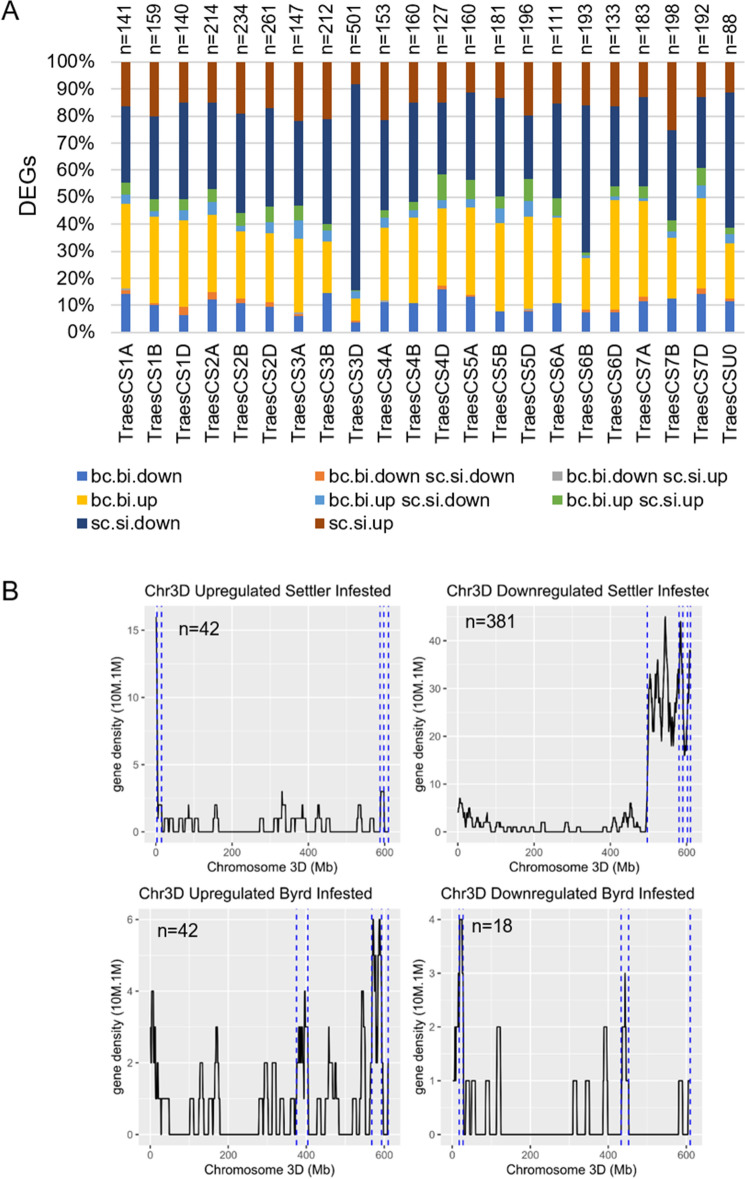
DEGs space organization. (**A**) Repartition of the 4084 DEGs on the 21 wheat chromosomes. bc, Byrd control; bi, Byrd infested; sc, Settler CL control; si, Settler CL infested. (**B**) Plot density of the DEGs on the chromosome 3D. Gene density was represented in a window of 10 Mb with a sliding window of 1 Mb. n indicates the number of genes. Blue dash lines separate the different segments identified with changepoint.

Among the 501 DEGs located on the chromosome 3D, 381 (76%) genes were downregulated in Settler CL after WCM infestation (Fig. 4A). Of these 381 genes on the chromosome 3D, 333 genes were located in the telomeric part of the large arm of the chromosome 3D (Fig. 4B). The functions of these 333 genes were linked to protein N-Linked glycosylation, phytochromobilin metabolic and biosynthesis processes, and positive regulation of stomatal complex. Among the top 10 genes with the highest fold-change, two genes were not expressed in the infested condition of Settler CL and expressed in the uninfested conditions: *TraesCS3D03G0974000LC* (Protein FAR1-RELATED SEQUENCE 5) and *TraesCS3D03G1005800LC* (60S ribosomal protein L5) (Supplemental Table 2). Other genes had functions related to GDSL esterase/lipase, Proline-rich protein, Germin-like protein 1–1, Dirigent protein, Arginine decarboxylase, or Oxidation resistance protein 1 (Supplemental Table 2).

### Hierarchical clustering exhibited gene function enrichment specific for each wheat variety

**Figure 5:**
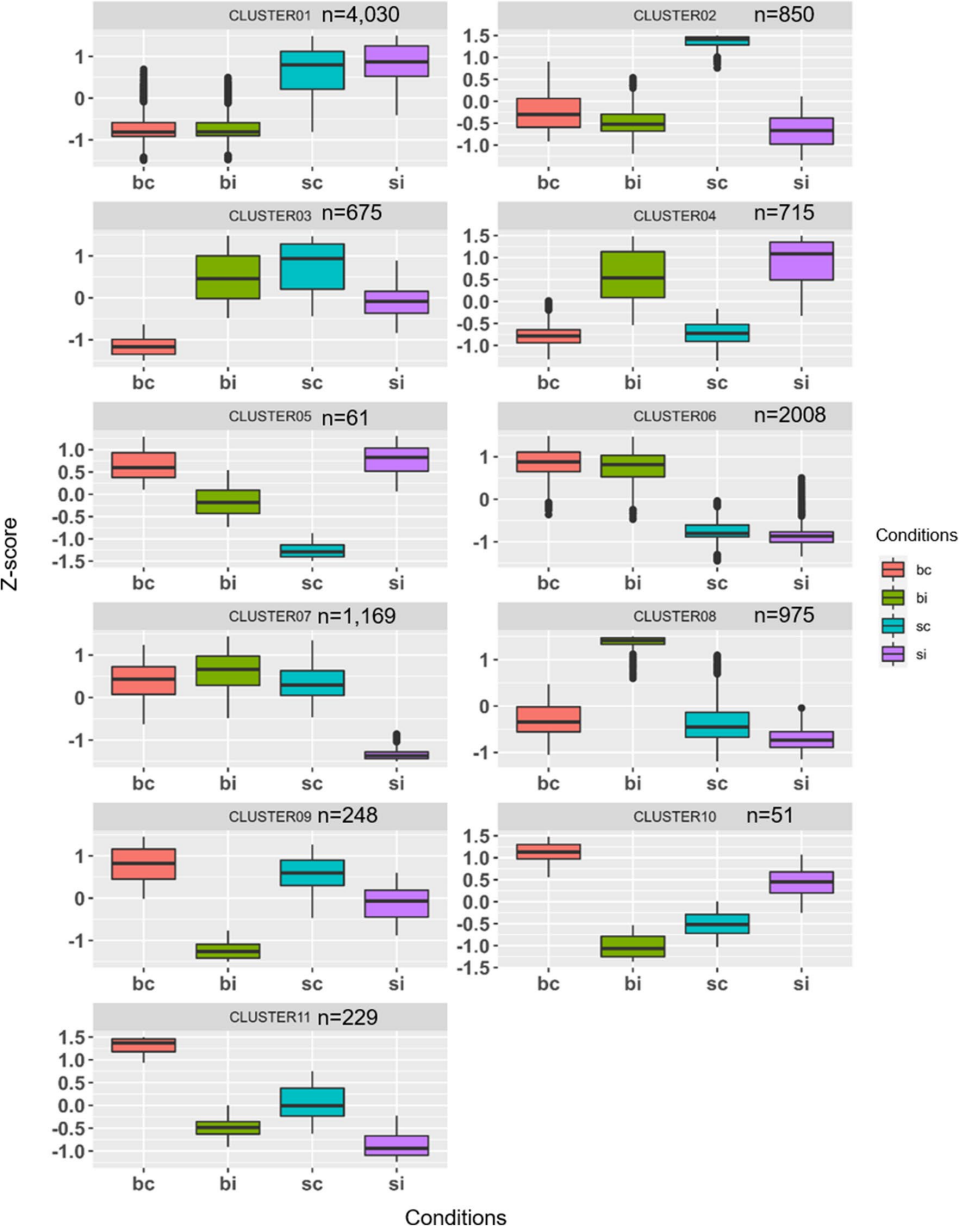
Expression profiles of the DEGs for the 11 clusters. Expression values are given in Z-score (FPKM-mean _FPKM_/standard deviation _FPKM_) (red = bc, green = bi, turquoise = sc and purple = si). bc, Byrd control; bi, Byrd infested; sc, Settler CL control; si, Settler CL infested.

Overall, 11,016 DEGs displayed up or downregulated between all the conditions. Clustering patterns of DEGs under WCM infestation were determined by hierarchical clustering analysis of all DEGs. The 11,016 DEGs were grouped into 11 clusters that included from 51 (cluster 10) to 4030 (cluster 1) genes (Fig. 5). Clustering analysis showed genes activated in the infested and uninfested conditions (cluster 1) of Settler CL were related to lipid transport and localization and protein methylation and alkylation (Supplemental Table 4). Alternatively, cluster 6 contained genes activated in the infested and uninfested conditions of Byrd with functions related to protein transport and localization, and maintenance of cellular protein location. Genes activated in the uninfested condition of Settler CL were grouped in cluster 2 (850 genes), with functions related to stomatal development. The genes in cluster 3 (675 genes) comprised of those activated in the infested condition of Byrd and uninfested conditions of Settler CL, and these genes were deactivated in uninfested Byrd and the infested conditions of Settler CL. The 675 genes had functions related to response to hormones, stress, and endogenous stimuli. Genes activated after WCM infestation in both Byrd and Settler CL were grouped in cluster 4 (715 genes) and had functions related to transport of metal ions and cations. However, cluster 11 genes (229 genes) were activated in Byrd and Settler CL controls and deactivated in infested Byrd and Settler CL with functions related to lipid transport and localization, lipid phosphorylation, RNA methylation. Cluster 9 genes (248 genes) were highly deactivated in WCM-infested Byrd and activated in Byrd and Settler CL controls, and these genes had functions related to sucrose metabolic processes. In cluster 7 (1169 genes), genes were deactivated only in WCM-infested Settler CL, and these genes had functions related to carbohydrate catabolic process, polysaccharide catabolic and metabolic processes, lipid transport and localization, and responses to drugs. Genes with functions related to asparagine synthase, catalytic activity, protein dimerization or ligase activity were part of the cluster 8, where genes were activated in WCM-infested Byrd plants (Supplemental Table 4).

### Variety-specific metabolic pathway response to WCM infestation

**Figure 6:**
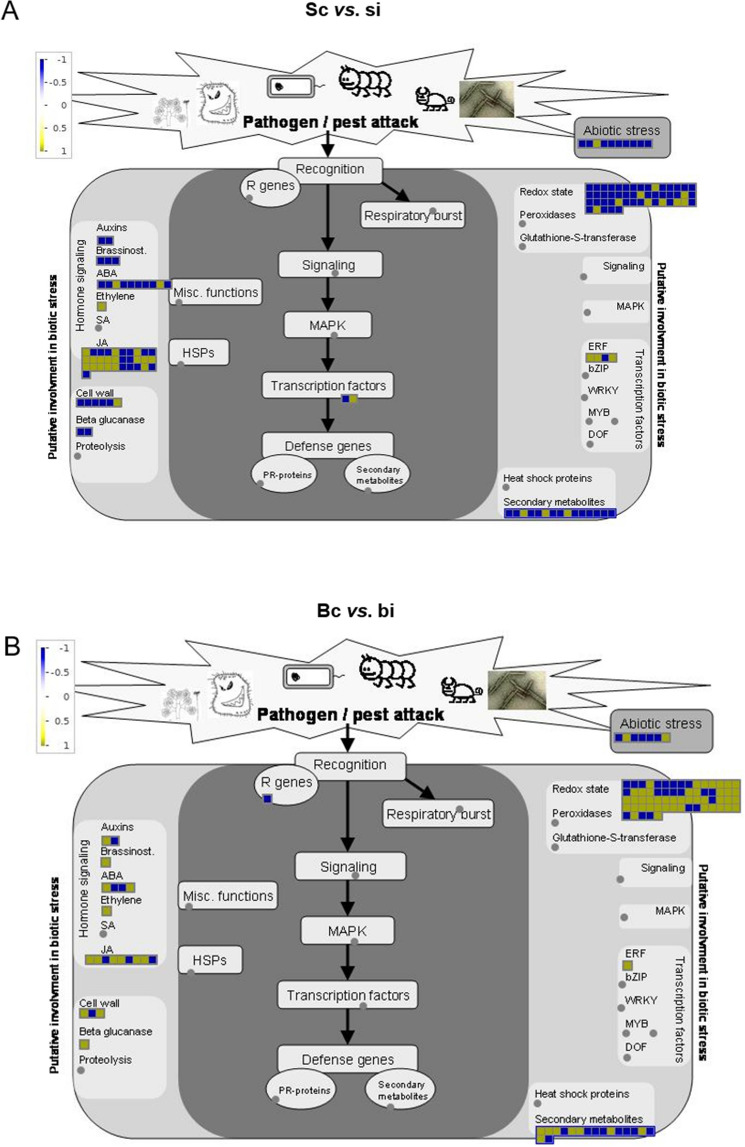
Overview of the transcriptomic response after WCM infestation using Mapman for the susceptible cultivar (**A**) and resistant cultivar (**B**). Each box represents the − log_10_ (FC). Yellow indicates upregulated gene expression and blue downregulated gene expression in response to WCM. bc, Byrd control; bi, Byrd infested; sc, Settler CL control; si, Settler CL infested.

We used Mapman to investigate the variation of the metabolism pathways and processes in both cultivars in response to extended feeding by WCM. Our results indicate that more pathways related to cell wall, secondary metabolites, redox states, or hormonal pathways (e.g., JA and ABA) were detected in the susceptible cultivar at 10 dpi (Fig. 6A). This could explain the higher number of DEGs observed for the susceptible variety. However, genes involved in these pathways were mainly downregulated in the susceptible variety (Fig. 6A). The number of genes related to metabolic pathways in the resistant cultivar were low, but these genes were mostly upregulated (Fig. 6A). Further, upregulated DEGs in the resistant variety were involved in hormonal pathways such as JA and ABA, redox state, or cell wall biosynthesis (Fig. 6B).

## Discussion

Timely induction of defense signaling mechanisms contribute to a robust defense against insect attack^40–42^. Leaf curling resulting from WCM feeding has been used to score wheat varieties as susceptible or resistant against WCM^23,34^. At 20 dpi, visual differences were detectable between leaves of susceptible and resistant varieties. Susceptible plants displayed longitudinal leaf curling and leaves trapped within the curl of older leaves (Fig. 1). However, symptomatic leaves are not easily noticeable until leaves were heavily infested with WCM. Leaf rolling or curling has been described in crops leaves in response to various stresses such as salt, drought, and WCM^25,43^. A few genes have been involved in these leaf morphological changes such as, *LCR* (*LEAF CURLING RESPONSE*)^44^, TaDUFF699 gene family^45^, *OsLBD3-7*, *NLR1,* and *ACL1*^46^. These previous studies demonstrated the modification of the plant transcriptome linked to changes in leaf morphology. The reduction of curling symptoms in resistant varieties could potentially help decrease the total number of WCM in the field due a less favorable niche on the plant and increased exposure to other abiotic and biotic factors not present in our controlled lab experiments.

Wheat transcriptome responses to short-term feeding (1 dpi) by WCM has been recently reported and provided insights about early defense mechanisms utilized by wheat against WCM^38^. However, WCM has a rapid reproductive rate, with egg-adult developmental times of 7 to 10 days^47^. Thus at 10 dpi, the plant will be interacting with the next generation of WCM. While symptoms on wheat leaves are not strong at 10 dpi, the impact of WCM feeding that leads to curling had been started, thus we investigated the variation of the transcriptomic response between resistant and susceptible wheat varieties after extended feeding (i.e., 10 dpi). In their study, Kiani et al.^38^ used TAM 112 (WCM-resistant cultivar) and Karl 92 (WCM-susceptible cultivar). TAM 112 has two WCM-resistant genes (*Cmc3* and *Cmc4*), and it is one of the parent lines of Byrd that has one WCM-resistant gene (*Cmc4*)^33,48^. The parental link between the two cultivars allows for a global comparison between these studies.

The number of DEGs for each cultivar between WCM-infested and uninfested conditions were higher after 10 dpi compared to short-term (1 dpi) feeding^38^. Our results showed the proportion of upregulated DEGs were higher only in the resistant cultivar. At 10 dpi, we also observed a higher number of downregulated genes in the susceptible cultivar compared to the susceptible cultivar at 1 dpi as seen by Kiani et al.^38^. These results suggest that the early defense mechanisms that were activated during WCM infestation were transient. At 10 dpi, 134 genes were upregulated in the resistance cultivar and downregulated in the susceptible cultivar. The functions related to these genes showed the importance of the production of stress-related hormones and structural components in biological membranes. Phospholipase A1 genes catalyzes the hydrolysis of fatty acids and the release of alpha-linolenic acid, which has been described as a JA precursor^49^. The role of fatty acids in plant defense has been characterized for the response to fungal and insect infections^50^. Fatty acid levels will increase insect elicitor induced defense response^51^. Fatty acids also have a role in wax composition which represent a physical barrier for insect/pest feeding^52–54^. In our experiment, after 10 days, WCM affected leaf morphology by preventing the leaves from unfolding and by consequence proper leaf development. The alteration of the leaf morphology in the susceptible cultivar at 10 dpi affects stomatal development, which could lead to the alteration of the photosynthesis and plant development. The action of WCM on resistant cultivar at 10 dpi was related to primary nitrogen metabolism with the inactivation of asparagine synthase genes that play an important role in nitrogen assimilation and distribution. Nitrogen also plays a role in gene transcription by its involvement in RNA synthesis^55^. Collectively, our data suggest that the wheat transcriptome was impacted at 10 dpi in the susceptible variety.

Plant resistance can be separated into three resistance categories: antibiosis, antixenosis, and tolerance^56^. Tolerance is the plant’s ability to withstand or recover from insect/pest damage; however, the mechanisms underlying tolerance are poorly understood. Recent studies have suggested that phytohormones play a major role in plant tolerance to insects^57–59^. Previously, it was shown that metabolite levels were altered in wheat plants after short-term (1 dpi) feeding by WCM^38,60^. While there were many differences in the responses between susceptible and resistant varieties, comparing DEGs between 1 dpi^38^ and 10 dpi highlight some important mechanisms that can contribute to strengthening host plant resistance. First, we observed that DEGs related to cell wall composition in the resistant variety were downregulated after 1 dpi^38^, but they were upregulated after 10 dpi. This indicate that resistant varieties are able to maintain a cell wall structure after prolonged WCM feeding. Second, phytohormones play key roles in herbivore-induced defenses by activating key early signal transduction pathways^61^. Our study identified a high number of DEGs involved in JA and ABA that can potentially modulate WCM-induced stress responses. ABA is a phytohormone that regulates plant growth and development, and abiotic stress responses in plants^62^. ABA did not show significant induction at 1 dpi in either susceptible or resistant varieties. In contrast, genes related to ABA were downregulated in susceptible varieties but upregulated in resistant varieties at 10 dpi. We hypothesize that ABA will accumulate in the resistant wheat in response to damage of bulliform cells and photosynthesis and respiration stress from early symptoms of leaf curling. JA also plays an important role in plant response to biotic stress. DEGs related to JA pathway in susceptible varieties did not show significant response at 1 dpi yet they had a mix of both up- and downregulation of genes at 10 dpi. However, genes related to JA pathway was upregulated in resistant varieties at both time points. This suggests that JA plays a major role in wheat defense against WCM. A previous study on hexaploid bread wheat (*T. aestivum* var. Zhongmai 175) infested with phytotoxic and non-phytotoxic aphids for two days exhibited the induction of plant defense responses in addition of JA, SA, and ET pathways^63^. The expression of genes involved in phytohormone pathways in wheat is triggered by various insects/pests, and this process occurs after short and longer exposure to insect feeding.

Chemical defenses play a decisive role in induced defense mechanisms against herbivore infestation^64^ We saw downregulation of genes related to secondary metabolites in the susceptible wheat variety, but a clear pattern for the resistant variety was not seen at 10 dpi. Interestingly, DEGs related to secondary metabolites were upregulated at 1 dpi in the susceptible variety^38^. Five DEGs at 10 dpi had functions related to chymotrypsin inhibitor. Trypsin and chymotrypsin are the major digestive serine proteases in lepidopteran insects. In Arabidopsis, transgenic expression of barley protease inhibitor genes provided enhanced resistance to spider mites (*T. urticae*)^65^. No investigations had been yet performed on the gut composition of WCM feeding on wheat. However, our results suggest a role of chymotrypsin inhibitor in the wheat resistant cultivar at 10 dpi, possibly by countering WCM gut/saliva secretion. Together, these results highlight the defense mechanisms used by the resistant wheat cultivar to limit WCM colonization. Plant resistance against insect herbivory has focused on antibiosis, but evolution and adaptation of target pest population is inevitable. Focusing on mechanisms that contribute to plant tolerance would be a more sustainable strategy. We believe further studies can benefit from exploring the genetics of morphological features of tolerance (i.e., reduction of curling symptoms) and physiological mechanisms (e.g., ABA affecting photosynthetic rate, growth rate post infestation).

Because of its large genome size (17 Gb), wheat gene space organization was characterized with high gene density in the telomeric chromosome area^66,67^. The investigation of the location of the downregulated genes in the susceptible cultivar were only found enriched in the telomeric area of the large arm of the chromosome 3D. *Aegilops tauschii* has been identified as a donor of the D-genome for the allohexaploid wheat (*Triticum aestivum* L.)^68^, and the D sub-genome contains fewer genes than the A and B sub-genomes. Nevertheless, the D-genome has been identified as a reservoir for biotic and abiotic stress tolerance, and *A. tauschii* has been used to transfer useful genes to the allohexaploid wheat by direct hybridization or synthetic wheat for pest/pathogens resistance, abiotic stresses, and quality traits^69,70^. The geographical origin of *A. tauschii* in arid and semi-arid areas has been linked with the drought resistance role of the genes carried by the D-genome. The morphological changes of the leaves in response to drought stress is similar to the response to WCM for wheat. This could result from the inactivation of genes located in the sub-genome D. In this study, downregulated genes were related to stomatal complex development, tissue development, and phytochromobilin. These functions in leaves are responses to drought for water retention (reduction in stomata density, low transpiration efficiency, and increased stomata size)^71^. Byrd resulted from the crossing of C0970547-7 and TAM112^48,72^. The crossing history of TAM112 included an *A. tauschii* line, TA2460, known to carry the leaf rust resistance gene *Lr41* and origin of the *Cmc4* gene^27^. This information attests of the importance of the D-sub-genome in resistance to WCM. The investigation of the function of all the genes located in the 3DL telomeric region revealed functions related to plant defense mechanisms. Interestingly, among the gene set located on 3DL and downregulated in the susceptible cultivar, 10 genes are upregulated in the resistant cultivar at 10 dpi. These 10 genes are part of the 134 DEGs mentioned previously and have functions related to defense (*TraesCS3D03G1115200*: Phospholipase A1) or cell wall fortification (*TraesCS3D03G1196300*: Hydroxycinnamoyl-CoA transferase 2; *TraesCS3D03G1035400*: Hydroxyproline-rich glycoprotein). Further investigation will be necessary to evaluate the transcriptomic activity of these genes during a time course in the wheat resistant cultivar. Thus far, WCM resistant genes *Cmc4* and *Cmc2* have been identified in chromosome 6D of hexaploid wheat^27,33,34^. In this study, we have shown that a portion of chromosome 3D was deactivated after prolonged WCM feeding in the susceptible cultivar. These results will be an additional resource for plant breeders to develop wheat resilient cultivars and increase the genetic diversity.

## Conclusions

In this study, we provide evidence of defense mechanisms used by a resistant wheat variety containing the *Cmc4* gene against WCM after extended feeding. Action of phytohormones, combined with lipid signaling and membrane integrity play a role in response to WCM after 10 dpi. A higher number of molecular functions are activated at 10 dpi compared to 1 dpi^38^ in the resistant variety. In addition, the importance of the genes located in the sub-genome D of the wheat in response to mite feeding is identified.

## Materials and methods

### WCM population maintenance and infestation

The study was conducted using Type 2 WCMs^16^. The mite colony was maintained on ‘Settler CL’ (NH03614) wheat plants in 15-cm diameter pots with plastic cylindrical cages. The cage had two, 8-cm diameter openings covered with Nitex® screen (80-micron mesh opening; BioQuip Products, Rancho Dominquez, CA) on opposite sides one-third the way from the bottom. The colony was maintained under artificial light with a 14:10 (L:D) photoperiod at 22–24 °C. Mites were transferred to new wheat plants in pots every four weeks to maintain the colony.

To perform infestation, only active adults (ca. 190–255 μm) displaying normal movement were used. Mites were transferred with the aid of a dissecting microscope (magnification ca. 30-40X) by using a single human eyelash glued to a wooden dowel to transfer individual mites. Ten mites were selected and released onto a small paper isosceles triangle (1 cm height). The triangles were then placed into the whorl of 2- to 3-leaf stage (14 days after planting) healthy wheat plants.

### Plant materials and samples collection

Two hard red winter wheat varieties (*T. aestivum* L.) were used, Byrd and Settler CL. Byrd is a WCM-resistant wheat variety^48^ and Settler CL is a WCM-susceptible variety^73^. Seeds were planted individually in cone-tainers (4 cm top diameter and 20 cm length) with standard greenhouse mix. These cone-tainers (Steuwe and Sons Inc., Corvallis, Oregon, USA) were covered with tube cages and kept in the growth chamber with 14:10 (L:D) photoperiod at 25 °C and ca. 60% relative humidity. Cages were made from clear cylindrical plastic tubes (5 cm diameter and 50 cm length), vented with three 5-cm diameter openings covered with Nitex® screen.

At 14 days after planting, wheat plants were checked for uniformity in phenotypic growth and health before being used for WCM infestation. The study was conducted as a randomized complete block design with a factorial arrangement of treatments consisting of two wheat varieties (WCM-resistant and WCM-susceptible) and two WCM treatments (infested and non-infested). For each treatment, three replicates were used, and for each treatment, a replicate consisted of three individual wheat plants. At 10 dpi, whorl tissue samples were collected. Tissue sampling consisted of collecting leaf whorl tissue (ca. 3 cm) from each of the three plants per treatment and replicate into a single sample and flash-freezing the tissues in liquid nitrogen.

### Nucleic acid extraction and mRNA-seq library construction

Wheat whorl tissues (80–100 mg) were ground using 2010 Geno/Grinder® (SPEX SamplePrep, NJ, USA) for 40 s at 1400 strokes min^−1^. Total RNA was extracted from the homogenized tissue using the kit NucleoSpin miRNA for miRNA and RNA purification (Macherey–Nagel, NucleoSpin miRNA, Mini kit for miRNA and RNA purification, ref 740,971.50). Extracted total RNA was quantified through Nanodrop 2000c Spectrophotometer (Thermo Scientific TM). Then, stranded mRNA-seq library construction and sequencing (Illumina) was commissioned to Genewiz (South Plainfield, USA). mRNA-seq libraries were sequenced in 150 bp paired-end with 20 million reads on average per library.

### Transcriptomic analysis

The quality check of the RNA-seq libraries was performed with FASTQC^74^ and reads with a Phred score lower than 20 and length below 45 base pairs were removed with Trimmomatic v0.39^75^. Then, trimmed reads were mapped on the wheat reference genome v2.1 (https://wheat-urgi.versailles.inra.fr/Seq-Repository/Assemblies)^39^ with Tophat2^76^ using the following parameters: 2 mismatch (− N 2), 0 splicing mismatch (− m 0). The transcripts’ reconstruction was performed with Cufflinks v2.2.1 with the following parameters: quantification against the reference annotation only (− G), multi-read-correct (− u), and frag-bias-correct (− b). The differential expressed gene (DEG) analysis was performed with Cuffdiff 2.2.1. Differential expressed genes (DEGs) were identified with the following parameters: *P*-values ≤ 5% and false discovery rate (FDR) |log_2_(Infested/Contol)|≥ log_2_(2). All the statistical analysis were performed with R using the packages: stats^77^ and WGCNA^78^. Z-score were calculated with the formula FPKM-mean(FPKM)/sd(FPKM).

### Functional annotation

Gene ontology (GO) information was obtained from the IWGCS annotation v1 (https://urgi.versailles.inra.fr/download/iwgsc/IWGSC_RefSeq_Annotations/v1.0/). The GOBU package was used for enrichment calculations^79^. The full set of wheat gene annotation was used as the reference comparison set against down or upregulated DEGs. The *P*-values were calculated using Fisher’s exact test and corrected for multiple testing with the FDR method by using the R module called ‘*P*-adjust’.

### Segmentation/change-point analysis

Segmentation analyses were performed using the R package changepoint v1.0.6^80^ with Binary Segmentation method and BIC penalty on the mean change. The gene density was calculated in sliding windows of 10 Mb with a step of 1 Mb.

## Supplementary Information

Supplementary Information 1.

Supplementary Information 2.

Supplementary Information 3.

Supplementary Information 4.

Supplementary Information 5.

## Data Availability

The raw datasets generated during the sequencing of current study are available on this link: https://dataview.ncbi.nlm.nih.gov/object/PRJNA765290?reviewer=nc1ea48oagugv3v0ulpq7h8ih4.
