# Supplementary material for: Wheat transcriptomic responses to extended feeding by wheat curl mites
Source: Sci Rep. 2022 Jul 22;12:12535. doi: 10.1038/s41598-022-16792-1 (PMC9307608; doi:10.1038/s41598-022-16792-1)
Supplement: Supplementary file 5 — Supplementary Information 5. [file 41598_2022_16792_MOESM5_ESM.pdf]

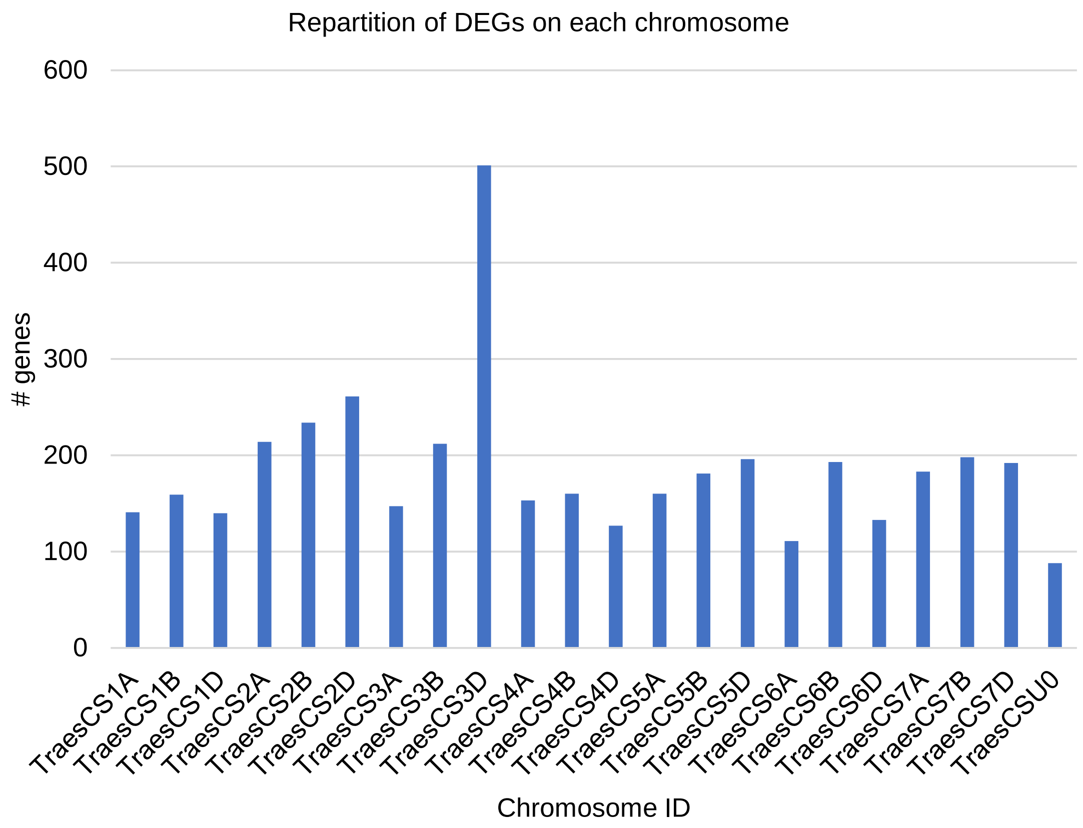

Supplemental Figure 1: Repartition of the 4,084 DEGs on the 21 wheat chromosomes. U0 indicates the scaffolds.

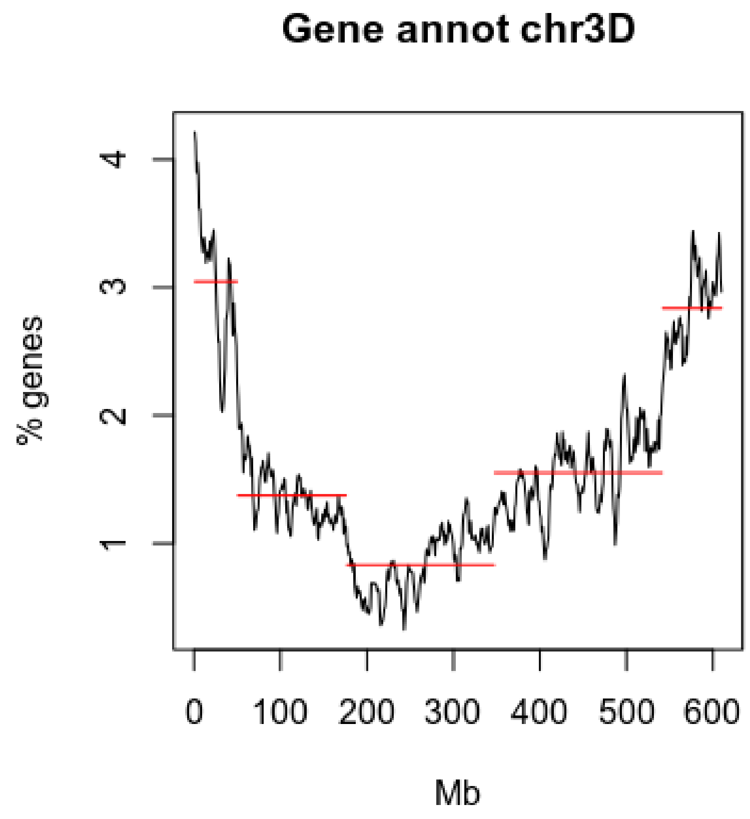

Supplemental Figure 2: Gene density of the annotation genes of the chromosome 3D. Red lines represent the segmentation.
